# Supplementary material for: Functional screening for anti-CMV biologics identifies a broadly neutralizing epitope of an essential envelope protein
Source: Nat Commun. 2016 Dec 14;7:13627. doi: 10.1038/ncomms13627 (PMC5171902; doi:10.1038/ncomms13627)
Supplement: Supplementary Information — Supplementary Figures 1 - 13 and Supplementary Tables 1 - 4 [file ncomms13627-s1.pdf]

Supplementary Figure 1

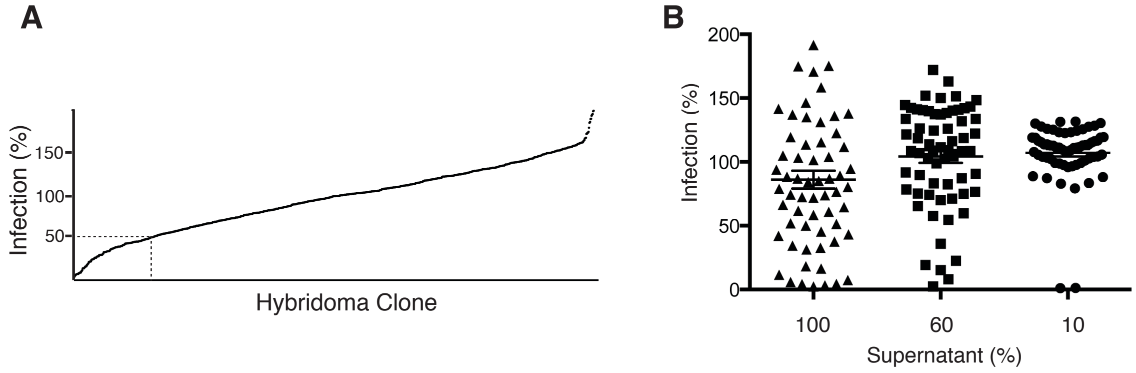

Identification of CMV-neutralizating antibodies by hybridoma screening. (A) Supernatants from ~2000 murine hybridomas were tested for their ability to neutralize CMV infection of MRC5 fibroblasts with AD169IE2-YFP following pre-incubation with virus. All samples were normalized as %infection compared to the median value for all samples. The dotted line represents the 50% infection cutoff used to select hybridomas for further screening. (B) Hybridoma supernatants from the clones that reduced infection by 50% were screened at 100%, 60%, or 10% hybridoma supernatant concentration and normalized to the median of all samples tested.

Supplementary Figure 2

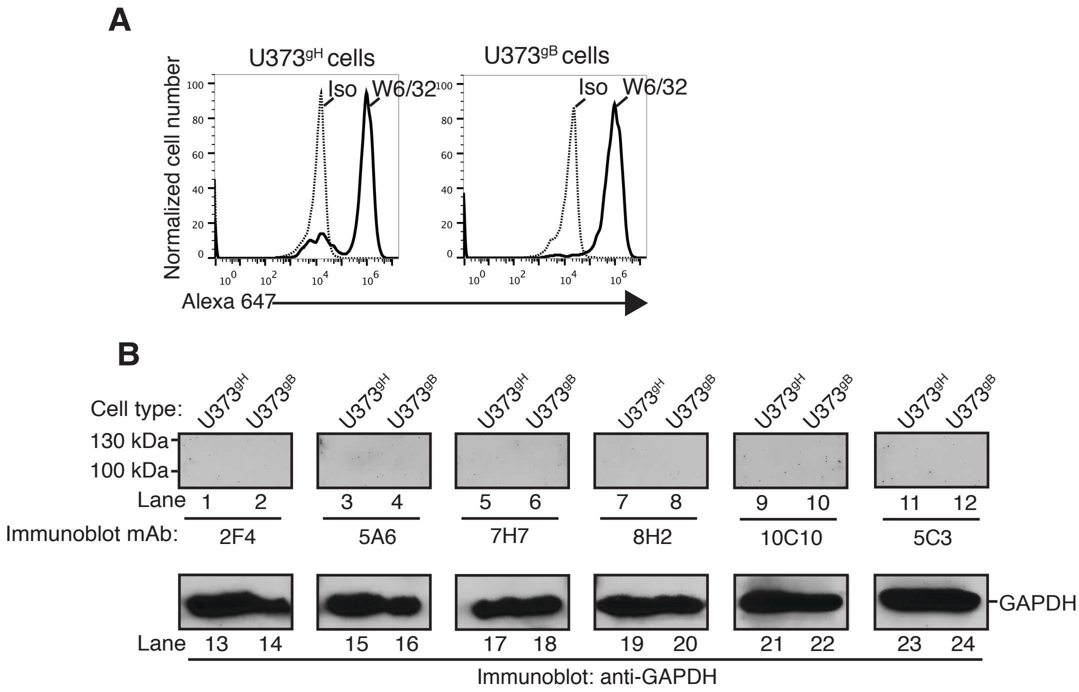

Examination of CMV neutralizing mAb targets. (A) U373gH (left panel) and U373gB cells (right panel) were permeabilized and stained with an IgG2a isotype control (dotted line) or an antibody recognizing properly folded MHC class I molecules (W6/32) (solid line) followed by flow cytometry analysis. (B) Lysates from U373gH or U373gB cells were resolved by SDS-PAGE and exposed to immunoblot with CMV-neutralizing antibodies (Lanes 1-12) or GAPDH (Lanes 13-24). Relative molecular mass markers are indicated.

Supplementary Figure 3

A

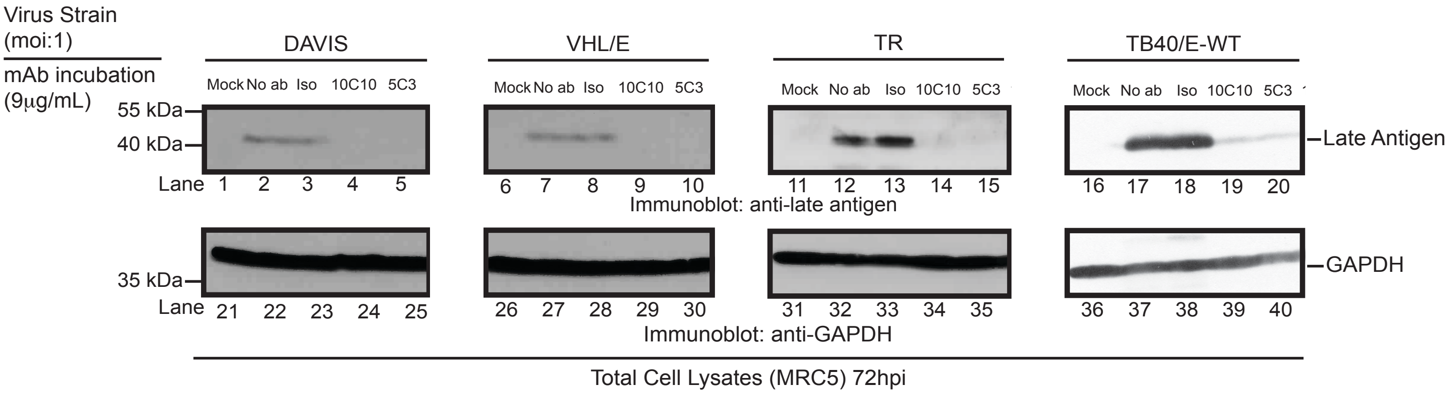

B

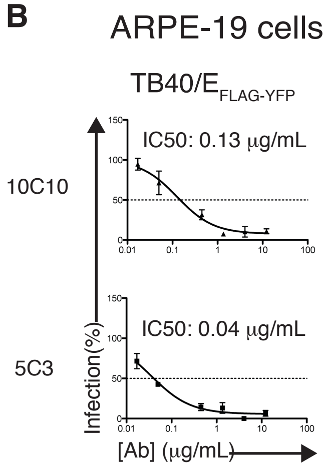

Neutralization of clinical-like CMV strains by anti-gH antibodies. (A) Total cell lysates from MRC5 cells infected with CMV strains DAVIS (first panel), VHL/E (second panel), TR (third panel), or TB40/E (fourth panel) following incubation with or without an isotype control or anti-gH mAbs 10C10 and 5C3 were resolved by SDS-PAGE and exposed to immunoblot for CMV Late Antigen (top row) or GAPDH (bottom panel). Relative molecular mass markers are indicated. (B) mAbs 10C10 and 5C3 were pre-incubated with TB40/EFLAG-YFP (.01-12μg/mL), and subsequent infection levels of ARPE-19 cells was measured by YFP fluorescence levels. IC50 values are indicated. Experiments for B were performed in technical triplicate and standard deviation is depicted.

Supplementary Figure 4

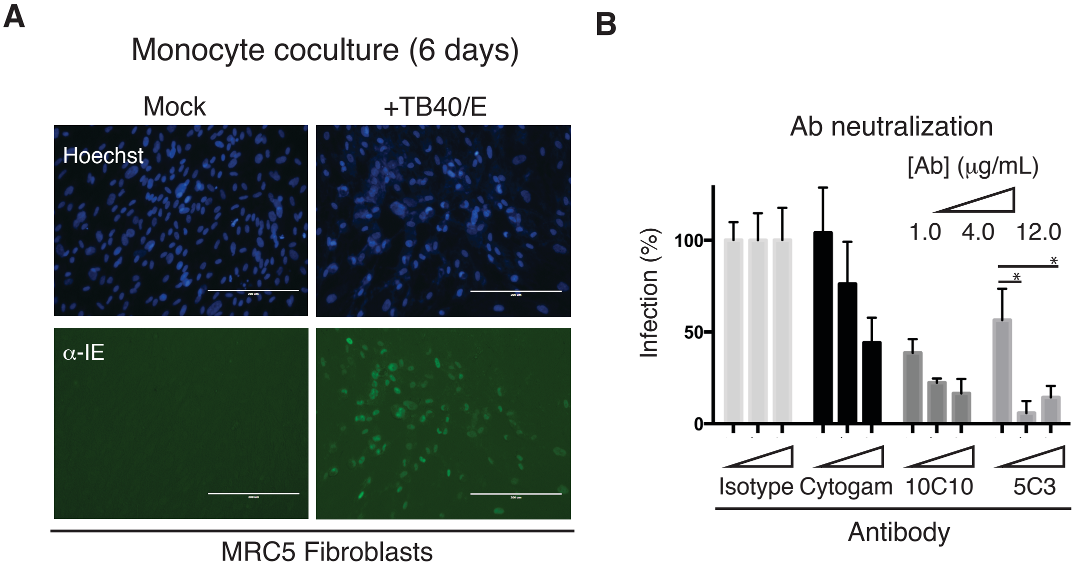

Anti-gH antibodies block monocyte-fibroblast dissemination. (A) Fibroblasts cocultured for 6 days with CD14+ monocytes that were either mock infected or infected with TB40/E were analyzed by fluorescence microscopy for presence of anti immediate early gene product (α-IEFITC) (bottom row). Hoechst reagent permitted visualization of the nucleus (top row). Scale bar = 200 micron. (B) The number of α-IEFITC -positive fibroblasts/well following coculture with infected or mock-infected monocytes in presence of various concentrations (1-12μg/mL) of Cytogam® or mAbs 10C10 or 5C3 was quantified by fluorescent cytometer. Error bars represent standard deviation and the data is averaged across three independent experiments. * = $p < 0.05$ (student's t-test)

Supplementary Figure 5

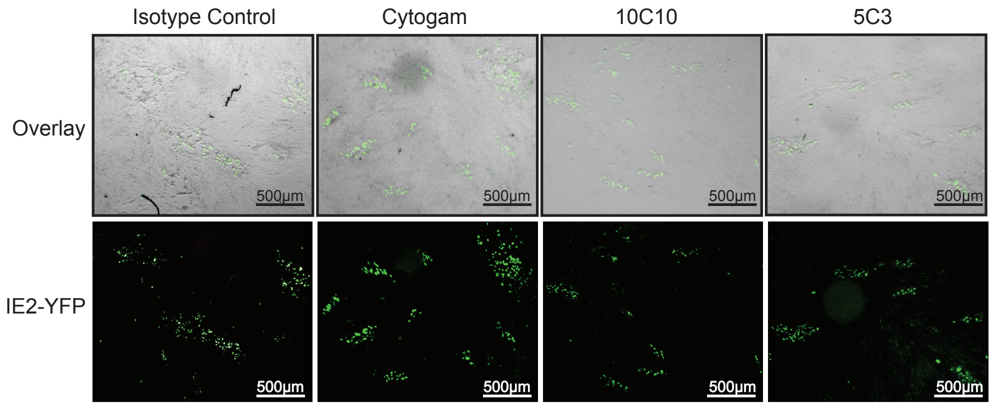

Anti-gH monoclonal antibodies limit viral dissemination following virus infection. MRC5 cells infected (moi: 0.1) with AD169$^{IE2/YFP}$ were treated with isotype, Cytogam, 10C10, and 5C3 (0.5μg/ml) at 24 hpi for up to 6 days. The cells were visualized (2X objective) using phase-contrast and fluorescence microscopy. A plaque was identified as a cluster of infected cells. The phase contrast and fluorescence signal were overlayed to identify a plaque and the number of infected cells within a plaque. Scale bar = 500 micron. The data demonstrates significant cytopathogenicity in the isotype antibody-treated cells and numerous virus infected cells/plaque. In contrast, treating cells with anti-CMV antibodies showed a decrease in cyto-pathogenicity suggesting an inhibition in viral dissemination. Infected cells treated with the anti-gH mAbs 10C10 and 5C3 demonstrated a decrease in both plaque number and virus-infected cells/plaque when compared to the Cytogam treated infected cells.

Supplementary Figure 6

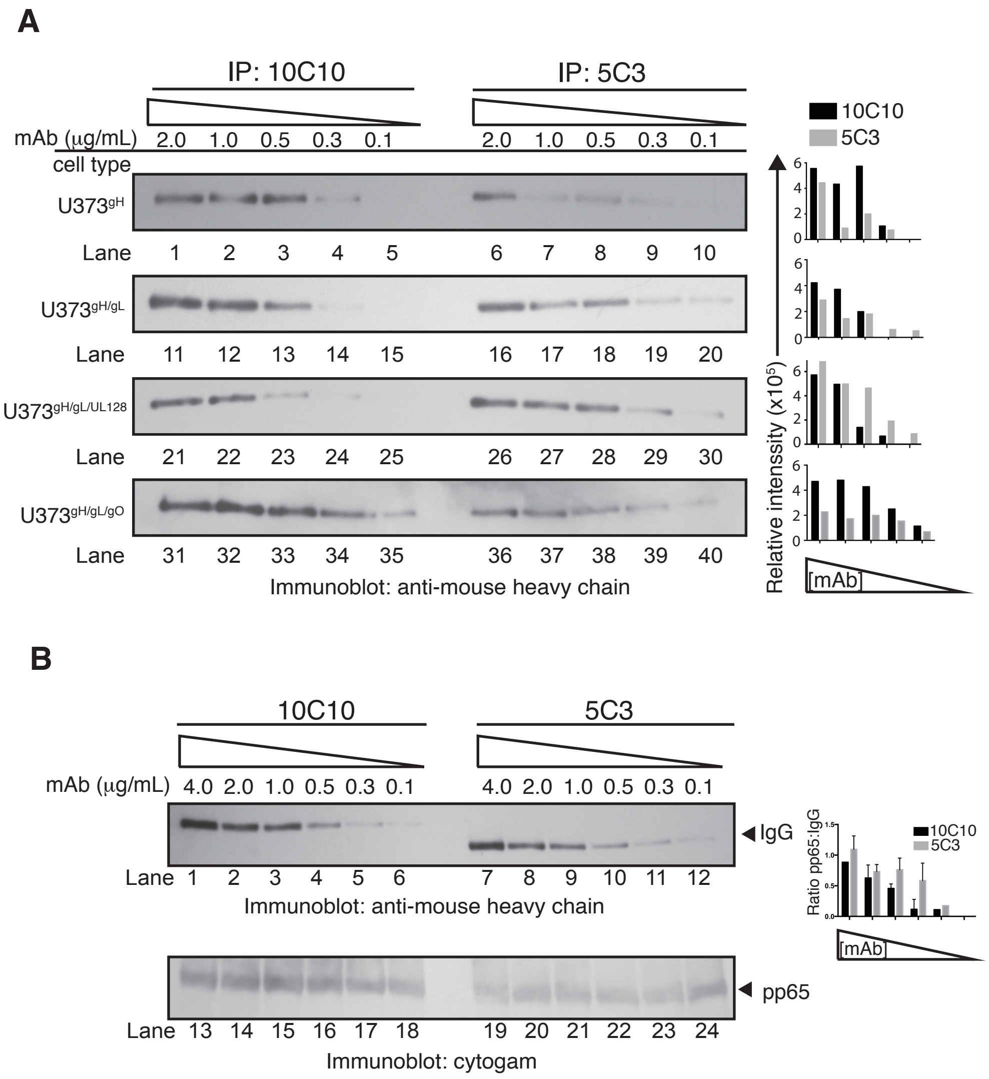

mAbs 10C10 and 5C3 display variable preference for gH protein complexes. (A) Lysates from U373gH (Lanes 1-10), U373gH/gL (Lanes 11-20), U373gH/gL/UL128 (Lanes 21-30), and U373gH/gL/gO-HA (Lanes 31-40) cells were exposed to 10C10 or 5C3 at various concentrations (0.1-2µg/mL) and the resolved immune complexes were exposed to gH immunoblot. Densitometry values of the recovered gH are depicted (right column). (B) TB40/E virus prep was incubated with varying concentration of mAb 10C10 and 5C3 (0.1-4µg/mL). Virus/mAb complexes were then recovered by ultracentrifugation and total protein was resolved by SDS-PAGE, followed by immunoblot for anti-mouse heavy and light chain (Lanes 1-12) or Cytogam® (Lanes 13-24). IgG and pp65 are indicated by arrows. Average densitometry values of the pp65:IgG ratio from 3 independent experiments are depicted (right column) with standard deviation.

Supplementary Figure 7

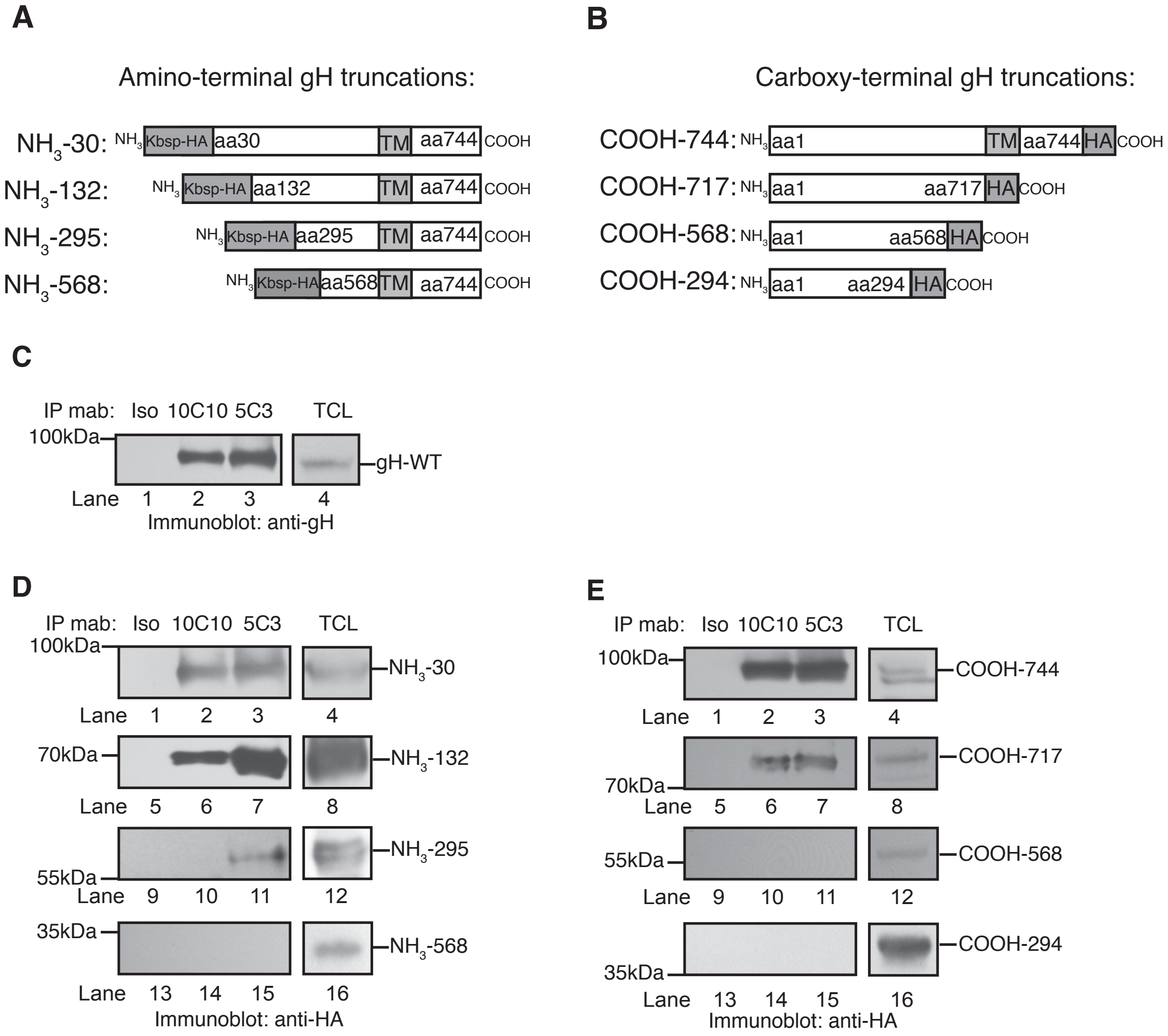

Binding of truncated gH constructs by mAbs 10C10 and 5C3. A series of gH N-terminal (A) and C terminal (B) truncation constructs were generated. (C) Conditions for optimal gH transfection and immunoprecipitation from 293 cells were established for the WT gH construct. Cell lysates from gH WT-transfected cells were exposed to an isotype control (lane 1) or mAbs 10C10 (lane 2) and 5C3 (lane 3). Recovered immune complexes were immunoblotted with an anti-gH protein. Total cell lysates demonstrated the proper expression of the gH protein (Lane 4). (D) gH N-terminal constructs and gH C-terminal constructs (E) were transfected into 293 cells. Total cell lysates were exposed to immunoprecipitation with an isotype control or mAbs 10C10 and 5C3. Immunoblot was performed with an anti-HA antibody. Total cell lysates demonstrated the proper expression of the gH mutant. Relative molecular mass markers are indicated.

Supplementary Figure 8

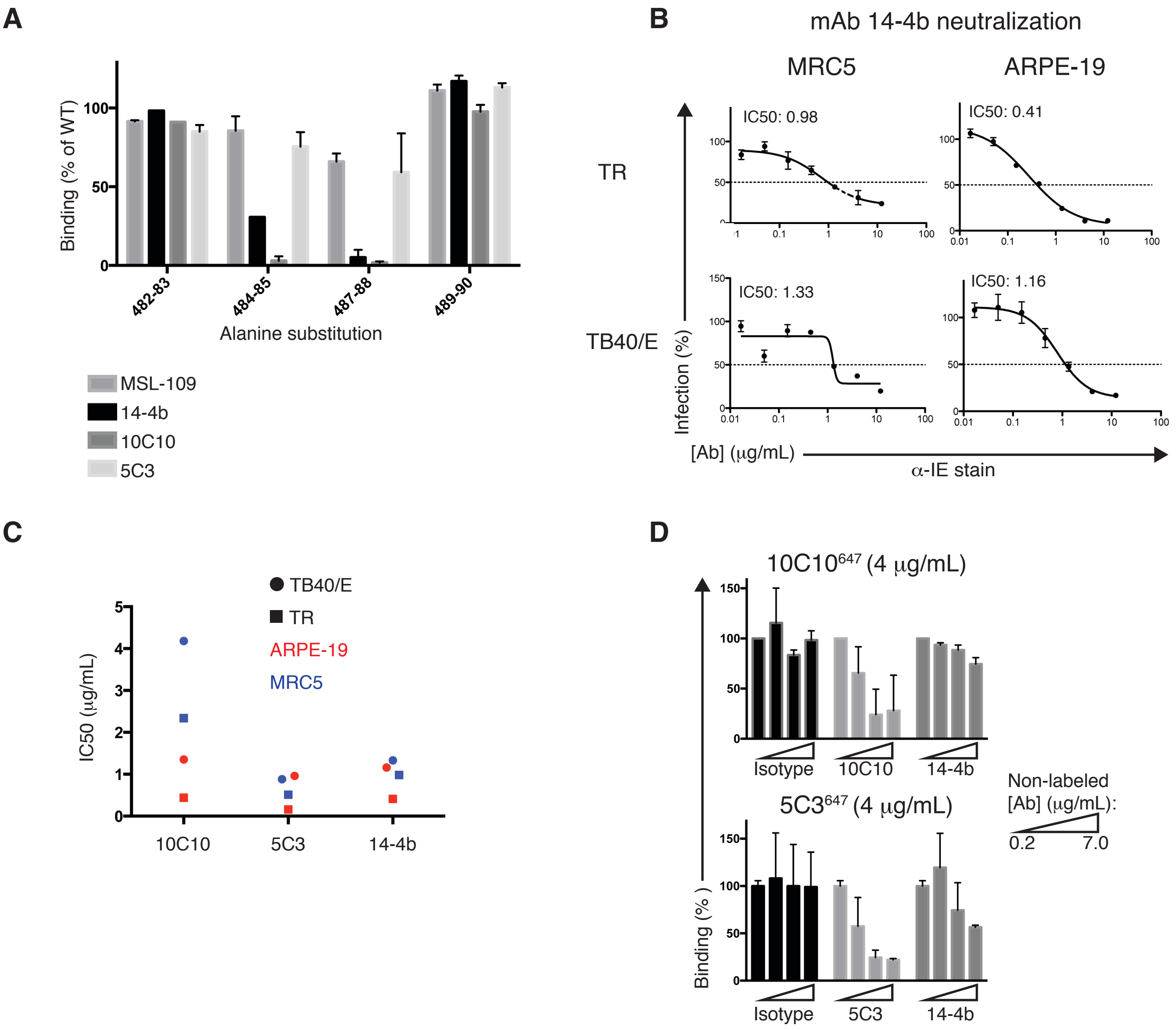

Characterization of neutralization and binding of the anti-gH mAb 14-4b. (A) HEK-293 cells transfected with gL and wild type gH or gH alanine mutants 482-83 (TT482-3AA), 484-85 (ER484-5AA), 487-88 (EI487-88AA), and 489-90 (FI489-90AA) were stained with MSL-109 (grey bars), 14-4b (black bars), 10C10 (dark grey bars), or 5C3 (light grey bars) and analyzed by flow cytometry. The % binding was determined using mean fluorescent intensity (three experiments) from wild type gH as 100%. (B) The mAb 14-4b was pre-incubated with CMV strains TB40/E and TR (.01-12µg/mL) before exposure to MRC5 or ARPE-19 cells. Cells were analyzed for virus infection at 16hpi using the anti-Immediate Early (IE) gene product (anti-IEFITC) antibody using an Acumen cytometer. Non-linear regression analysis was performed and the half maximal inhibitory concentration (IC50) was calculated for 14-4b. Experiments were performed in technical triplicate. Standard deviation is depicted. (C) The IC50 values of 14-4b, 5C3, and 10C10 were plotted based on infection of ARPE-19 and MRC5 cells and CMV strains TB40/E and TR. (D) U373$^{gH/gL}$ cells were were labeled with mAbs 10C10 (top row) and 5C3 (bottom row) conjugated to an Alexa^{647} fluorophore (10C10^{647} and 5C3^{647}(4µg/ml) with increasing concentrations of non-conjugated isotype, 10C10, 5C3, or 14-4b (0.2, 0.7, 2.1, and 7.0µg/mL) (in triplicate). The fluorescence signal from the labeled U373$^{gH/gL}$ cells was measured by flow cytometry. The % binding was determined using the mean fluorescence intensity measured from the corresponding isotype concentration as 100%.

Supplementary Figure 9

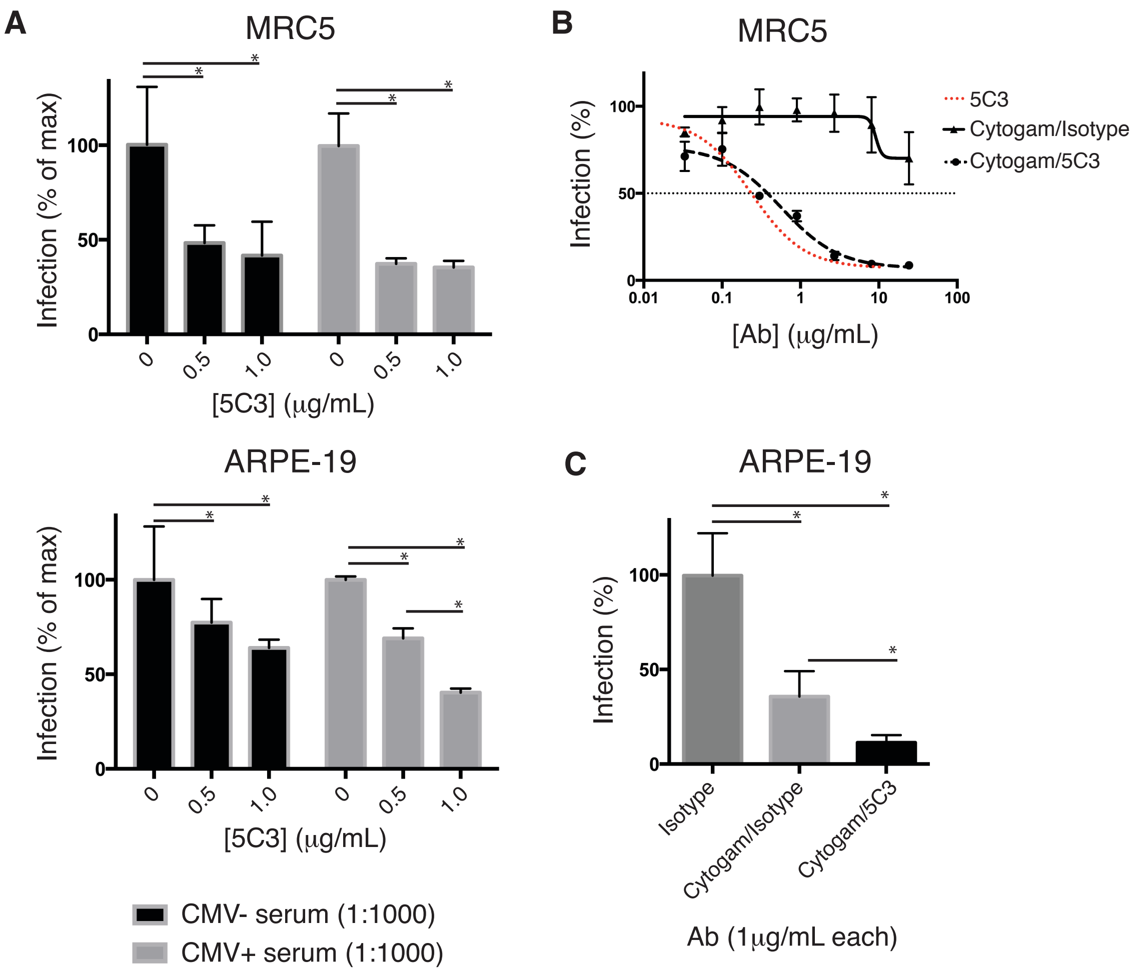

Anti-gH mAbs supplement pre-existing CMV immunity. (A) Serum from CMV-negative (black bars) or CMV-positive (gray bars) pregnant women was tested for its ability to neutralize a TB40/E infection with various concentrations of mAb 5C3 (0-1µg/mL) in MRC5 (top panel) or ARPE-19 (bottom panel) cells. (B) Cytogam® was combined with an equal concentration of an isotype control (solid line) or mAb 5C3 (dotted line), and pre-incubated with AD169IE2-YFP at multiple concentrations (.02-12µg/mL for each respective antibody). (C) Antibody cocktails containing 1µg/mL of Cytogam® with 1µg/mL of an isotype control, or Cytogam® with mAb 5C3 were tested for their ability to neutralize TB40/EFLAG-YFP infection of ARPE-19 cells. Experiments were performed in technical triplicate. Standard deviation is depicted for all experiments. * = $p<0.05$ (student's 2-tailed t test)

Supplementary Figure 10

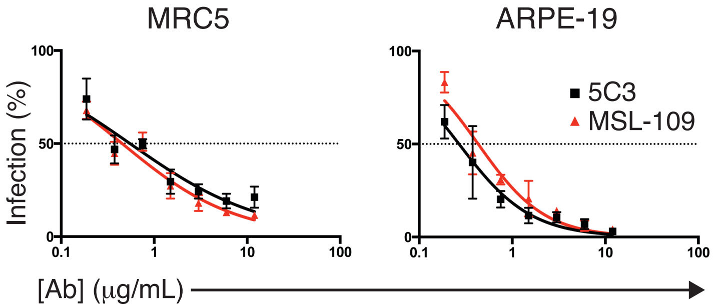

Neutralization by 5C3 compared to MSL-109. mAbs 5C3 (black) and MSL-109 (red) were pre-incubated with TB40/E at various concentrations (.01-12μg/mL), prior to exposure to MRC5 fibroblasts (top panel) or ARPE-19 cells (bottom panel). Subsequent infection levels were analyzed by immunostaining with anti-Immediate Early (IE) gene product (α-IEFITC) antibody. Infection levels were compared to an isotype control. Experiments were performed in technical triplicate. Standard deviation is depicted for all experiments.

Supplementary Figure 11

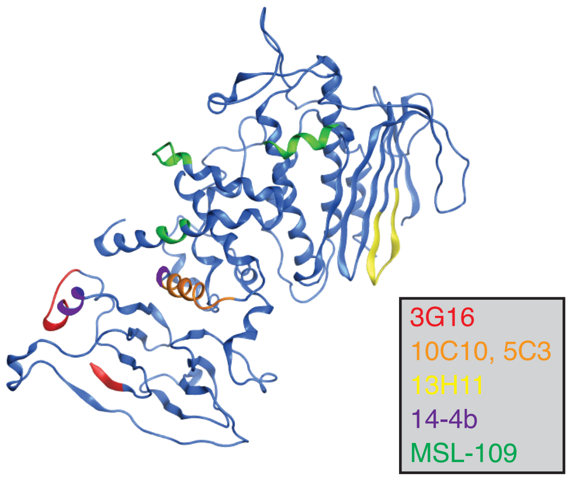

A 3D gH epitope map. A ribbon model of the predicted CMV-gH structure based on the crystalized HSV-1 gH/gL complex is depicted. The putative interaction sites of anti-gH CMV-neutralizing antibodies 3G16 (red), 10C10 and 5C3 (orange), 13H11 (yellow), 14-4b (purple),and MSL-109 (green) are shown.

Supplementary Figure 12

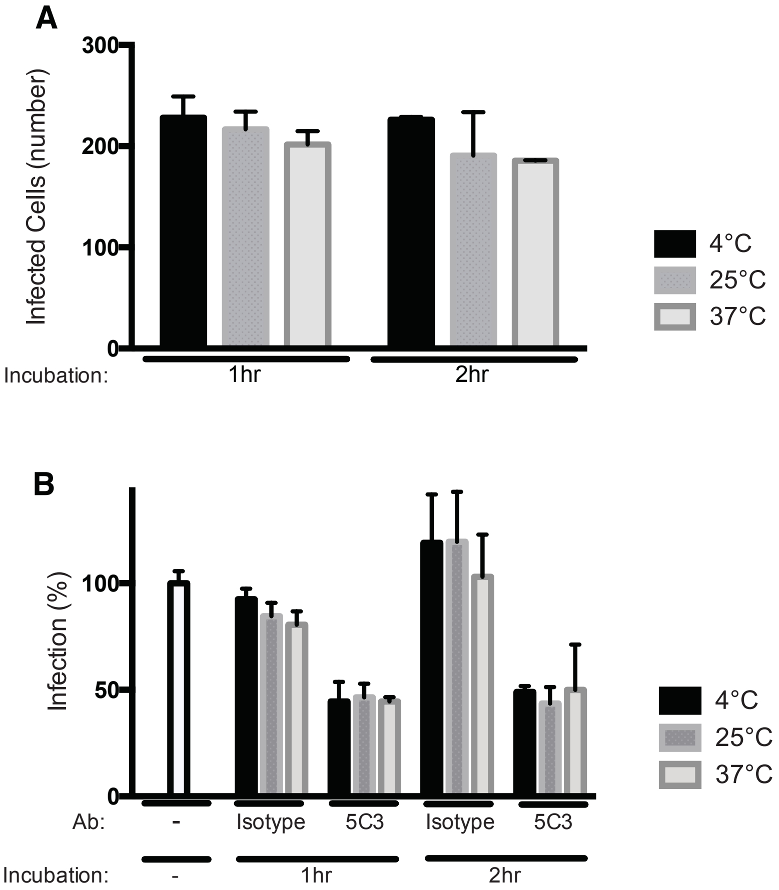

Determination of how temperature and incubation time affect virus infection and antibody neutralization. (A) MRC5 cells infected with AD169$^{IE2/YFP}$ (moi:3) pre-incubated for 1 or 2 hours at 4°C, 25°C, or 37°C (in triplicate) were analyzed for the number of infected cells (24hpi) using an Acumen cytometer for IE2-YFP positive cells. (B) MRC5 cells infected with AD169$^{IE2/YFP}$ (moi:3) pre-incubated for 1 or 2 hours at 4°C, 25°C, or 37°C with 5C3 or isotype control (EC50 concentration) (in triplicate) were analyzed for the number of infected cells (24hpi) using an Acumen cytometer for IE2-YFP positive cells. The % infection was determined using mock-treated AD169$^{IE2/YFP}$ without any incubation as 100%. Experiments were performed in technical triplicate. Standard deviation is depicted for all experiments.

Figure S13

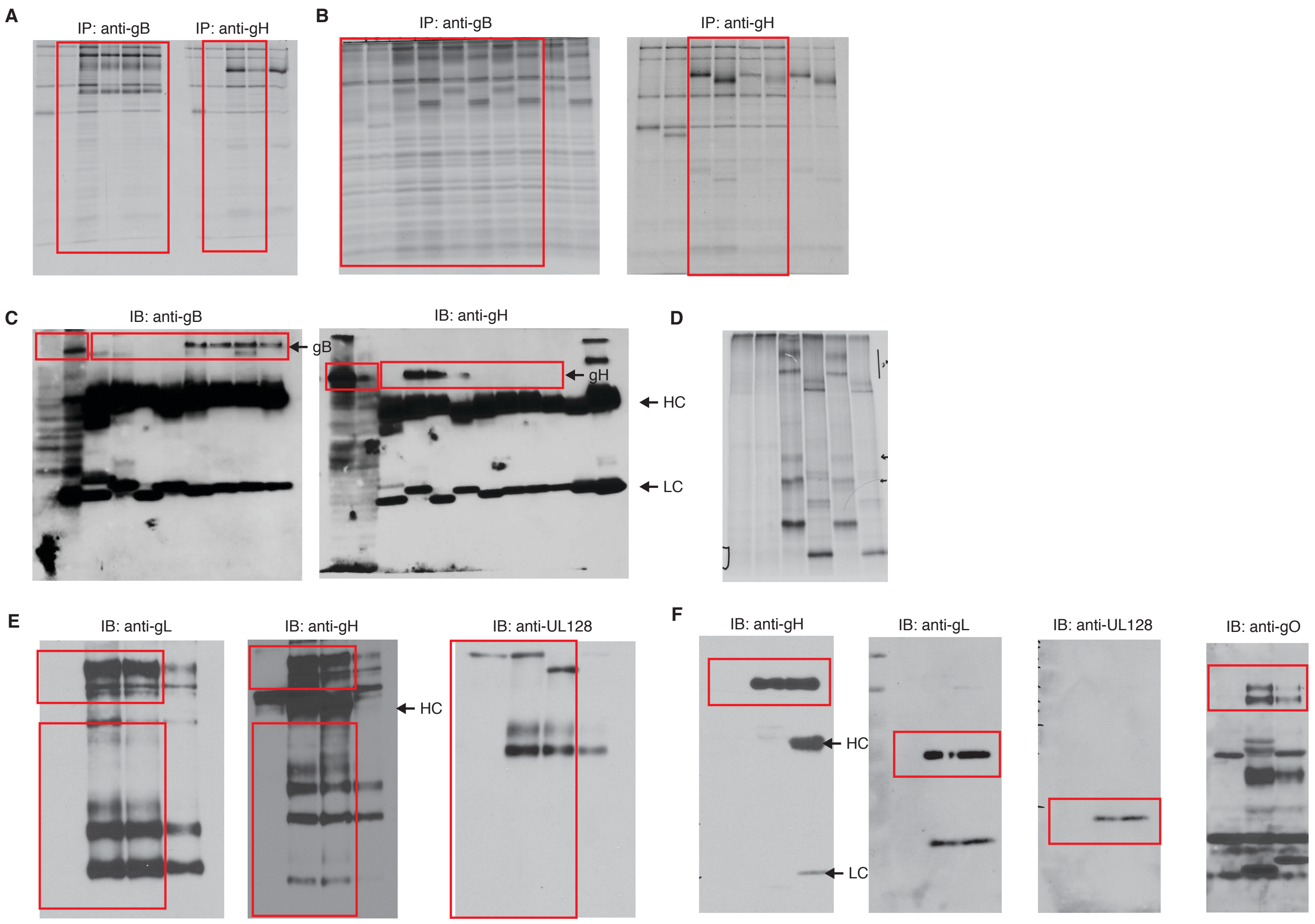

Uncropped Immunoblots and Autoradiographs (A) Uncropped autoradiograph from Fig. 2A (B) Uncropped autoradiograph from Fig. 2B (C) Uncropped immunoblots from Fig. 2E (D) Uncropped autoradiograph from Fig. 5A. (E) Uncropped immunoblots from Fig. 5B. (F) Uncropped immunoblots from Fig. 5C. HC - immunoglobulin heavy chain, LC - immunoglobulin light chain.

Supplemental Table 1: Major peptide species precipitated by CMV-neutralizing mAbs

Ab Group	Clone	Molecular weight (kDa) (approx.)	Rank	Log(I)	Gene	Protein
1	2F4	130	1	7.8	UL55	gB
2	10C10	100	1	8.01	UL75	gH
		30	1	7.74	UL115	gL

Supplementary Table 2: IC50 values of anti-gH mAbs compared to Cytogam® (µg/mL)

**Strain**	**AD169-IE2-YFP**	**TB40/E FLAG-YFP**	**TR**			**TB40/E**			**VHL/E**			**DAVIS**
**Cell Type**	**Fibroblast**	**Fibroblast**	**Fibroblast**		**Epithelial**	**Fibroblast**		**Epithelial**	**Fibroblast**		**Epithelial**	**Fibroblasts**
**Assay**	**IE2 fluorescence**	**FLAG-YFP fluorescence**	**Plaque**	**α-IE**	**α-IE**	**Plaque**	**α-IE**	**α-IE**	**Plaque**	**α-IE**	**α-IE**	**Plaque**
**10C10**	0.5	0.38	2.43	2.34	0.44	1.28	4.18	1.35	0.24	0.99	0.11	0.37
**5C3**	0.24	0.07	2.13	0.63	0.16	0.45	0.88	0.96	0.02	0.51	0.11	0.22
**Cytogam®**			>6.25	>12.15	1.58	>6.25	>12.15	6.86	0.12	>12.15	10.91	>6.25

Supplemental Table 3: Summary of mass spectroscopy analysis of viral proteins associated with gH from an anti-gH (10C10) immunoprecipitation

Protein Name	Gene name	Description	E-value	Intensity	Peptides	Coverage (%)
Q69155	UL75	Envelope glycoprotein H	-485	9.09	18	36.33
A8T7J6	UL115	Envelope glycoprotein L	-169.6	8.81	8	37.1
D2W9Z9	UL128	Envelope protein UL128	-123.3	7.77	3	23.97
A0A0G2U4X8	UL130	Envelope glycoprotein UL130	-42.9	8.09	4	28.04
Q8AYZ3	UL74	Envelope glycoprotein O	-26.5	7.9	4	8.66
A0A0G2TCY2	UL131A	Envelope protein UL131A	-24.5	7.83	2	18.6

Supplementary Table 4: Relative binding of gH alanine substitution mutants

	Avg. binding (% of max)		
**Alanine mutant**	**MSL-109**	**10C10**	**5C3**
480-86	66	3	14
485-92	65	2	49
478-88	100	100	100
480-81	87	70	72
482-83	92	91	85
484-85	86	3	76
486-87	66	2	59
488-89	111	98	113
490-91	89	72	92
